# Multidisciplinary team functioning and decision making within forensic mental health

**DOI:** 10.1108/MHRJ-01-2018-0001

**Published:** 2018-09-10

**Authors:** Alina Haines, Elizabeth Perkins, Elizabeth A. Evans, Rhiannah McCabe

**Affiliations:** 1 Health Services Research, University of Liverpool, Liverpool, UK; 2 Centre for Innovative Ageing, College of Human and Health Sciences, Swansea University, Swansea, UK; 3 Northumberland Tyne and Wear NHS Foundation Trust, Newcastle upon Tyne, UK

**Keywords:** Decision making, Forensic mental health, Service user involvement, Multidisciplinary team (MDT) meetings, Team functioning, Video observations

## Abstract

**Purpose:**

The purpose of this paper is to investigate the operation of multidisciplinary team (MDT) meetings within a forensic hospital in England, UK.

**Design/methodology/approach:**

Mixed methods, including qualitative face to face interviews with professionals and service users, video observations of MDT meetings and documentary analysis. Data were collected from 142 staff and 30 service users who consented to take part in the research and analysed using the constant comparison technique of grounded theory and ethnography.

**Findings:**

Decisions taken within MDT meetings are unequally shaped by the professional and personal values and assumptions of those involved, as well as by the power dynamics linked to the knowledge and responsibility of each member of the team. Service users’ involvement is marginalised. This is linked to a longstanding tradition of psychiatric paternalism in mental health care.

**Research limitations/implications:**

Future research should explore the nuances of interactions between MDT professionals and service users during the meetings, the language used and the approach taken by professionals to enable/empower service user to be actively involved.

**Practical implications:**

Clear aims, responsibilities and implementation actions are a pre-requisite to effective MDT working. There is a need to give service users greater responsibility and power regarding their care.

**Originality/value:**

While direct (video) observations were very difficult to achieve in secure settings, they enabled unmediated access to how people conducted themselves rather than having to rely only on their subjective accounts (from the interviews).

## Background

The concept of multidisciplinary working in the UK is not new and in mental health care has been written about since the 1970s. In 1984 the Department of Health’s “Planning for the Future” strategy recommended the establishment of multidisciplinary psychiatric teams (MDT) comprising psychiatrists, psychiatric nurses, psychologists, social workers and occupational therapists to provide comprehensive treatment and care for people with mental health problems (Study Group on the Development of the Psychiatric Services, 1984). Based on the assumption that MDTs can improve the quality of care by including the perspectives of these professionals into the service users’ planning of care (Department of Health, 2007; Wagner, 2004), multidisciplinary working is now standard practice across all mental health services in the UK. “Effective and efficient multidisciplinary team working” is now the agreed approach for mental health services to address complex needs in severe mental illness (RCP, 2009).

A body of research evidence has been established to identify some of the benefits of MDT working within mental health services, such as reduced bed use (Marks *et al.*, 1994), improved service contact after discharge from hospital (Ford, 1995), preferred by service users and carers (Simmonds *et al.*, 2001), and improved job satisfaction amongst team members (Onyett and Ford, 1996). Underpinning the perceived strength of MDT working is the creation of a clinical environment that enables different professions to use their skills, knowledge, attitudes, values and abilities within their scope of practice (Orovwuje, 2008). However, as with the functioning of all teams, the operational reality of the MDT may be somewhat different, influenced by specific characteristics that make up a team (micro level), a ward/unit/type of security within a service (meso level), a mental health trust, with its policies and philosophy (macro level).

Forensic mental health services are concerned with the treatment and rehabilitation of service users who suffer from mental disorders and who pose, or who have posed, risks to others. The treatment within these services aims to manage the mental disorder at the same time as reducing the risk to others and offending behaviour, with the ultimate goal of a safe return into the community. Thus there is a strong emphasis on risk and security. To maintain safety, inpatients in secure care, whether high, medium or low secure services, are subject to a combination of physical, relational and procedural security. Relational security, which involves the therapeutic relationship between staff and patients and the mix up of staff, is a key concept within forensic services (Davies, 2004, p. 234). Risk and security are in a constant tension with therapeutic activities, and maintaining the right balance between the two is one of the biggest challenges in forensic mental health. In an increasing risk-averse society, taking therapeutic risks becomes difficult to achieve, which could lead to patients being detained for longer than needed (Davies, 2004, p. 234).

Professionals within forensic services are expected to manage risk and dangerous behaviour, but the power to predict future events with precision is limited, as there are many confounding variables that could influence these outcomes, beyond personality factors and psychotic ideation. Multi-disciplinary working and specialist skills are considered essential in understanding, assessing and managing risk and complex needs and delivering appropriate health care to these service users (Davies, 2004; Mason *et al.*, 2002).

While it is assumed that offenders suffering from mental disorders will benefit from the coordinated contribution of a wide range of professionals as they progress from secure care to rehabilitation in the community, this multidisciplinary collaboration may be difficult to achieve in practice (Shaw *et al.*, 2007). Sociological and organisational research studies indicate that effective interdisciplinary work requires more complex understanding and negotiating than just “the mere juxtaposition of professionals with different disciplinary expertise” (Liberati *et al.*, 2016). It has been argued that professionals’ attachment to existing boundaries (knowledge, skills, experience) can hinder communication and coordinated working at the expense of poor care integration and patient safety (Atwal and Caldwell, 2002; Oborn and Dawson, 2010; Ferlie *et al.*, 2005; Mackintosh and Sandall, 2010; Waring *et al.*, 2015). Previous research also indicates that not all mental health workers are keen or have the necessary skills to work effectively in teams (Singh, 2000) and that the lack of clarity and confusion regarding individual roles, leadership, clinical accountability and understanding between professionals making up the team can also be an issue (Burke *et al.*, 2000).

A recent systematic review of the functions of MDT meetings in community mental health care (Nic a Bháird *et al.*, 2016) highlights that research regarding the way in which multidisciplinary collaboration can actually be achieved in practice is limited. The authors conclude that there is a need for a “clearer understanding of MDT functioning” if best practice were to be achieved across mental health services (Nic a Bháird *et al.*, 2016, p. 133). This paper aims to address this gap by exploring team functioning and MDT decision making within forensic settings; while acknowledging that the way in which MDTs operate within forensic mental health is different from community or other acute settings. The legal context, and in particular the importance given to risk within the decision-making process, are key to understanding the operation of MDTs within forensic settings.

This paper is based on an ethnographic study commissioned by a mental health trust in England to investigate the operation of MDT meetings within a medium secure forensic hospital. The format of MDT meetings was changed following a major enquiry into the management and discharge of a patient who subsequently committed a serious offence. The enquiry raised concerns about the hierarchical, non-participatory nature of meetings which were dominated by an overly clinical agenda. As a result, the MDTs within this service were restructured to address specific recommendations designed to improve effectiveness through a more democratic, transparent approach to decision making. The new MDT meetings were expected to be convened in accessible venues; conducted within structured agendas led by an independent chair; with minuted attendance and equal input from key professionals; and more involvement from service users, including the support of Independent Mental Health Advocates. This paper focusses on the functioning of the “reformed” MDT meetings and the way in which treatment decisions were made within this context.

## Methods

### Research design

This study used mixed methods, including face to face semi-structured interviews, non-participant observations and document analysis. Purposive sampling was used to obtain a sample of respondents from each discipline (present and/or contributing to MDT meetings) and from the five wards at the clinic (four 12-bedded male wards and one 8-bedded female ward).

### Data collection

In total 142 staff and 30 service users consented to take part in the research. Of these, 20 staff (3 chairs; 4 psychiatrists; 4 psychologists; 1 social worker; 2 occupational therapists; 6 nurses) and three service users were interviewed. The interviews were audio recorded and transcribed. In addition, 19 meetings were observed; 16 meetings were video recorded and 3 audio recorded. Approximately 38 per cent of MDT meetings taking place during the ten week period of data collection were observed. The observations covered all the wards and responsible clinicians (RCs) within the clinic. Each observed meeting discussed between one and three service users with an average time spent of 32 minutes per service user; 39 service users were discussed in total.

### Data analysis

The constant comparison technique of grounded theory and ethnography (Glaser and Strauss, 1967; Charmaz, 2006) was used to analyse the data. Observation notes and interview transcripts were scrutinised to identify common themes or variants on themes, which were refined in a continuous iterative process and validated by three researchers. The observation and interview data sets were initially coded independently of each other and the interview analysis was used to develop a checklist for the analysis of the observations. Data were analysed to explore similarities and differences between disciplines. The observations were analysed using the focussed observation technique (Angrosino and Mays dePerez, 2000), which allowed observations to be recorded in a narrative manner but within a structure. The structure was based on the emergent themes of the interview data as well as the format of MDT meetings so that what happened, how it happened, when it happened and its consequences were recorded. This included individual contributions (written, verbal and non-verbal) to discussion and decision making, the dynamics and interaction within the meetings and the meeting outcomes. In an iterative process, observational, interview data and documents were considered together to confirm, challenge or clarify findings.

### Ethical considerations

Following ethical and research governance approvals (REC reference 13/NW/0519; R&D reference 2013/22), participants were recruited and gave consent to be interviewed and/or to be observed during their usual meetings. To maintain confidentiality, participants’ names were removed from the data. In the quotes included here, participants will be referred to by their discipline or role (CH = Chair; RC = responsible clinician, which in all cases is a psychiatrist; PSY = psychologist; OT = occupational therapist; SW = social worker; Nurse = Nurse; Patient = P; and a randomly allocated number). To be noted that patients are referred to as “service users” throughout the text.

## Findings

### MDT meetings in practice

Decision making in the observed MDT meetings was a complex process and needs to be understood in relation to the way in which the team meetings operated at the time of data collection, especially with regards to the changes to the format of the meetings instituted as a result of an enquiry triggered by a serious incident. Table I captures a summary of the findings from the interviews regarding the nature and extent of these changes.

Each MDT was managed by a consultant psychiatrist – the RC – who had a caseload of between 12 and 15 service users. The teams met every week for approximately four hours and every service user was discussed every two weeks. On average, nine (6–12) professionals attended the meetings, e.g. the chair, the RC, a medical trainee, a social worker, a psychologist, an OT, a nurse and/or a ward manager, and admin support. On occasions, a dual diagnosis practitioner and student nurses also attended. The service user under review was also invited to attend at the end of the meeting. Attendance was often dictated by room location and/or size, as not all meeting rooms were fit for purpose, especially those on the wards.

In all of the observations, the MDT meetings were chaired by a senior nurse (independent from the ward and the clinical decision making for the service user under consideration); structured around an agenda, with logged minutes and action points. There was an expectation that all MDT members would make an equal contribution, first through a summary prepared in advance, then through input to emerging discussion points. Attendance at the MDT meetings was not mandatory. This is surprising, given that the meeting was the only opportunity for the team to make decisions with regards to a service user’s care. Attendance by profession was variable. Psychologists only attended half of all the observed meetings which affected discussions on risk formulation and understanding progress within a psychological approach to recovery. Just over two thirds of meetings were attended by occupational therapists and social workers which affected understanding of the social aspects of a service user’s progress. Thus, as certain team members were not present, the gap in knowledge meant that discussion was stunted and decisions could not be made. Service users were only invited to participate at the end of each meeting, to be provided with feedback on what has been discussed and the opportunity to ask questions. In nearly three quarters of the observed meetings a service user was in attendance.

In summary, the MDT meetings attempted to incorporate the operationalization of the care plan (long term), the review of the patient’s presentation (two weeks/short term) and progress taking into account the views of all disciplines present, as well as having service user input within a 30 min slot. In less “complex” cases, this was possible to achieve. However, the observations ranged from 16 minutes to 60 minutes, with the longest time being taken where the case was complex and the service user attended.

Having provided an image of the structure and conduct of the MDTs, in the next section of the paper we direct our attention to three key factors which influenced the way in which the observed MDT meetings functioned and shaped the decision-making process. These included: the way in which team members viewed the team in terms of their own professional roles and responsibilities; the MDT members’ attitudes towards risk and the management of disagreement; and the way in which the service user patient’s voice was heard, but limited their involvement in decision making.

### Professional roles and responsibilities

The way in which the concept of a team was interpreted by the participants in this study varied. This variation was underpinned by each professional’s view of their roles and responsibilities. In the interviews, for example, working in a team raised issues of loyalty for the nurses: loyalty to the team vs loyalty to the nursing profession:

I think […] I’ve always felt that we use that word team, but – how can I put it? It’s like a confederation in the sense that the loyalty lies to the discipline, so a nurse’s loyalty lies to the nursing discipline. Psychologists to their discipline and then they come together as a “team” to give their feedback but it’s like an association where, yes we’re just coming together to work but my real team is my discipline

(CH3).

This perception of having divided loyalties suggests that the professional identity of the nurse is deeply rooted and difficult to override merely by locating the individual in the context of other professional groups. Both in the interviews and in the observations there was also a sense in which the presence of other professions strengthened the nurses’ need to create boundaries around their contribution to the decision making. One of the psychologists reported the way in which nurses lay claim to be “knowing” the patient in a unique and exclusive way as an example of the lack of understanding team members had of other people’s roles in the MDT:

So when I am saying something in ward round, and I am using nurses as an example, they say no, all that doesn’t bother, they completely disagree with it, what’s he on about? You are not here day to day, you don’t see it day to day; no I don’t, but sometimes it is easier to observe the game from the stands than playing. So yeah, I think sometimes we don’t understand each other’s roles in the process, but sometimes I think people forget their own roles as well. So it can make it difficult. On my ward there is a particular issue around a patient and the nursing staff feel that the team, because sometimes I feel the nursing staff feel separate from the team

(PSY18).

Other members of the MDT supported the idea that different professions had views of the service user based on different types of knowledge:

Sometimes you get the sense of well we don’t see them every day like the nursing staff do, but sometimes we have a wider sort of picture because we see all the other things as well, so I do think we are valued in that sense that we do contribute

(SW6).

In a forensic mental health setting in which patients are involuntarily detained, decisions about care and treatment are framed by the twin paradigms of medicine and the law. Since many of the major decisions affecting the care of the detained patient relate to medicine within a legal framework, it is not surprising that RCs saw themselves as being directly accountable for decisions regarding the terms under which patients are detained, given leave, and discharged:

It is a forum and it’s a slightly kind of […] it’s somewhat skewed because a lot of the decisions only I can put my name to. So, if there are forms that need to be signed, then it always me that signs them, particularly around leave

(RC20).

In many ways this authority allowed clinicians to use their discretion in weighing up the value of the views of the other professionals in the meeting. The personality of the RC was identified as a key factor in determining the extent to which clinicians used this discretion to encourage other members of the team to contribute to the decision making:

[…] some of our Consultants are very open […] and are like welcome to ideas, and other consultants are […] will listen to you, but it’s my decision at the end of the day […] and there’s still that element of that going on

(Nurse16).

Interestingly, in the eyes of the RCs, the MDT meeting offered other professionals the opportunity to contribute to the decision making and placed a duty on other people to use that opportunity. While there was a consensus among nurses in the interviews and confirmed by the observations that everyone had input during the meeting, not all nurses felt that they were valued and listened to:

We’re a profession in our own right, we’re nurses, we do the day to day management on the ward. We’re the ones that have to carry out the MDT requests […] and I think sometimes it’s us that have to manage the risks more than any other MDT members, because it’s all supported at the front line, potentially firing line, for any aggression or violence and I think it’s vital that we’re listened to, and that we feel comfortable in carrying out what’s asked

(Nurse5).

This was also evidenced by the outcome of disagreements discussed in the next section. Interestingly, in contrast to the interview data, the observational data indicate that the opinions of nurses were considered and did have an impact on decision making.

### Attitudes towards risk and the management of disagreement

Unsurprisingly, risk was an important factor in decision making and was often the main focus of discussions. In fact risk often appeared to be the lens through which all MDT members examined an individual’s progress and treatment; and within the context of granting leave or discharge it became paramount.

In the interview data, risk aversion was not perceived to be a discipline specific trait but rather a characteristic of an individual. According to nurses, in face of disagreements or decisions regarding risk, “the hierarchy changes”, and the MDT working becomes less “collaborative” (Nurse10). A divergence between the reported views of staff who are not present within the MDT meeting as opposed to staff on the ward emerged:

When the staff are saying one thing, and some on the MDT are saying another, we have the conflict or their mixed discussion about what should happen. In my experience, the consultant or psychologist tend to win. I know win is the wrong word, but tend to have the final say […] Now I understand why the RC has to, because of the accountability factor […] but the same if it”s collaborative, is shared, it’s only shared to a point […] and when the bigger decisions are made, it then, in my opinion, doesn’t become shared

(Nurse10).

Interestingly, while nurses reported shifts in the hierarchy according to the decision to be taken, other team members suggested that, where nurses offer strong counter arguments to a proposed course of action, their views should be respected:

I think there’s far more appreciation given to the nurses’ perspective. Because it is the nurses that, you know it is recognised that the nurses will be the people who have to manage that decision on a day to day basis and over the twenty four hours. And so if they’re really sort of giving a strong argument perhaps against a decision being made, then that has to be acknowledged and accepted

(OT9).

Analysis of risk was often at the heart of disagreements between different professionals in the MDT meetings; and managing these situations was seen to be a very important aspect of maintaining the integrity of the team:

[…] the important thing there is to recognise that whatever we do there isn’t a right answer, and I think what’s important is that the team is cohesive enough to recognise that

(RC7).

[…] we’re thinking about the ethos of team work, and it would be that all opinions are discussed. But then there’s a common goal reached […] and even if someone didn’t originally agree with that, they champion it on behalf of the team

(PSY12).

This notion of “championing” decisions contrasts with the “grin and bear it” view of some of the nurses. In the observed MDT meetings the team most commonly deferred to the RC. However, there was much evidence of non-verbal disagreement in the form of eye-rolling; shaking heads; and looking around the room to gauge reactions of others.

### Hearing the voice of the service user

Results suggest that, in practice, the service users’ involvement was marginalised, and the views of the staff were at odds with the way in which service users actually participated in the meeting. Staff respondents reported the MDT meeting as service user-centred providing an opportunity to involve them in a discussion about their own care. But in reality, service users were invited to attend the MDT meeting at the end when everything had already been discussed and agreed upon. In the observations, the average time spent with service users was 5 min (with a range of 3 to 9 min) and, with the exception of one service user, there was little meaningful interaction. Although chairs and RCs made the effort to engage service users in conversation, their responses were often short and they seemed eager to leave after feedback:

It should be a forum for discussion with the service user and I think that’s probably an area that needs to be developed for them, it needs to be, you know, focussed on a bit more

(PSY14).

In the main, the team meeting provided an “audience” for service users to request things rather than an opportunity to discuss with the team their progress based on therapeutic milestones and their care plan:

If you discuss to most patients what [MDT] it’s about, it’s about getting leave […] Most patients aspects of well all we’ll get is leave, so that’s all I get from the MDT but it’s about planning where the next step is, where you’re aiming for, whether you’re going in to the community, are you going back home, are you going back to […] low secure

(Nurse1).

I worry sometimes that actually from the service user’s perspective, what it becomes is a [pause] an audience to request things’ […] “[…] can I have an increase in leave, can I have, you know. Can I have my clothes back, you know that sort of thing and that for me is not a purpose of someone”s involvement with the MDT, that’s not, not a helpful connection […] I think patients often don’t know what they have got to do to get to the next stage, it’s not clear for them you know

(PSY14).

Despite this, it was suggested that the MDT meeting is the only occasion a service user has an audience with all the care team members at one point in time:

It’s a forum for them rather than the staff discussing how things are going. And it is good for them because they really see, they see the doctor now and again, they see the psychologist once a week or something, they see us all the time; but it’s their opportunity to speak up for themselves

(Nurse17).

The service users interviewed mentioned building trust and relationships with their team as being important. From their perspective, attending MDT meetings provided an opportunity to “have a say”, to “find out where you are in the system” and especially to make requests, particularly with regards to leave. Contrary to what was observed, two of the respondents believed having an influence on the MDT decision making:

They take into account what I want, they understand where I’m coming from but they’ve gotta look at the risks and that as well

(P2).

In general decisions were made before the service users attended the meeting, leaving limited scope for service users to influence any outcome:

Yes, it’s not often where, you know, someone will come in, they’ve said they’ve not made a request for leave and then they’ve come in and said “oh I’d like this” and give us some reason. It triggers off a thought in the team who say “well leave it with us, we’ll talk about it” and if it’s reasonable, fine

(CH3).

This suggests that it is possible for service users to affect the outcome of decisions but that their concerns would most likely be discussed by the team without the service user present. There were no instances of this happening in the observations, but there were occasions in which service user’s requests were overlooked. In one such example, the medical trainee stated in the written summary that the service user wanted locality leave. However, this request was not discussed during the meeting and not brought up by the service user when he attended. Requests by patients for leave produced some of the most challenging discussions during the observational period. It is possible that as the request for leave by the patient was not repeated during the patient’s attendance at the MDT it was easier to behave as though the request had not been made.

While the trust included in this study supports collaborative working with advocacy services and IMHAs, the observations did not provide any evidence supporting advocacy attendance at the MDT meetings. Nurses, however, believed they had a role as “advocates” for service users:

[…] it can be quite intimidating […] as a named nurse you advocate for your service user anyway. So, as a nurse you would say […] “Do you want to go to your ward now?” “I don’t want to go” “Why not?” “Too many people in there. I know what they’re going to say to me already” “So what would you like me to say on your behalf?”

(Nurse16).

## Discussion

This paper used qualitative research methods to explore the functioning and dynamics of a multidisciplinary (MDT) decision-making forum in a forensic mental health hospital. Changes in the MDT meetings in the trust were implemented as a way of addressing concerns expressed in an enquiry about the process by which service users’ treatment and care was managed and discharges were made. The new MDT meeting was clearly reported to function differently from the way in which meetings had been previously run. However, there was some evidence that the new MDT meetings created or exposed some of the tensions that they were designed to overcome; namely boundaries between professionals in which team members report low team identification, but high professional identification (Onyett *et al.*, 1997; Brown *et al.*, 2000; Donnison *et al.*, 2009).

The majority of professionals were clear about their roles, but perceived that other members of the team did not recognise or understand these roles (Larkin and Callaghan, 2005). While the structure of the meetings was designed to promote the ethos of multidisciplinary work there was evidence that the professionals’ roles and responsibilities of the team members exerted an inescapable influence on decision making (Liberati *et al.*, 2016). In the context of forensic mental health provision this may not be surprising. Each professional group brings to the forensic domain their own knowledge and expertise, their profession’s value system, normative practices, ideological framework and ethical code of conduct. Additionally, each agency, clinic or ward has its own philosophy of care which may uniquely influence each profession’s value system. These local influences on practice in forensic mental health sit within the broader paradigms of medicine and the law. The medical model, with its focus on diagnosis and treatment, intersects with the legal paradigm with its articulation of the legal basis for detaining and treating people against their will. The dominance of these paradigms creates a framework within which the responsibilities for certain decisions are directly attributable to an individual, such as the RC. While other members of the team may contribute to a decision, the accountability for some decisions lies with a particular individual and so there will be circumstances in which the views of the other members of the MDT are marginalised or undermined (Chong *et al.*, 2013; Larkin and Callaghan, 2005; Onyett *et al.*, 1995). As Hudson (2002) suggests, where there is a clear hierarchy of professions, joint work may be more difficult because of the perceived status differentials, especially when bringing together professions such as medicine which might be consigned full professional status and other professions such as nursing and social work which might be associated with semi-professional status.

Although the intention behind MDT meetings was to provide an opportunity for all team members to report their views and contribute to decision making, it was clear that particular professional groups appeared to create boundaries around their area of expertise. (Fournier, 2000, p. 69) describes this “boundary work” as the product of two processes: “the constitution of an independent and self-contained field of knowledge as the basis upon which professions can build their authority and exclusivity and the labour of division which goes into erecting and maintaining boundaries between the professions”. Both nurses and RCs identified types of knowledge of the service user to which they had sole access. In the case of nurses, this was the day-to-day lives of the service user and in the case of the RC this was an overarching view of the service user in the clinical context of treatment and the legal context of detention. Since many of the major decisions being taken within the MDT meeting concerned leave and discharge, it was perhaps not surprising that the legal framework gave disproportionate control to the clinical members of the team who were deemed to be legally responsible for those decisions. Stacey *et al.* (2016) also reported in their study exploring shared decision-making in acute inpatient mental health care that, for psychiatrists, responsibility meant making the decision as opposed to facilitating shared decision-making.

Nurses frequently mentioned the importance of their “shop floor” perspective and that their insight into the recent presentation of service users should impact on decision making. As the implementation of team decisions often was the responsibility of nursing staff, a tension was created when they did not feel their voices had been heard. Although nurses reported that their opinions were often side-lined, this was not corroborated by observational evidence from this study. Whyte and Brooker (2001) found that the least qualified members who have the most face-to-face contact with service users often feel the most disenfranchised from the support and decision-making aspects of MDTs in secure environments. In this context the nurses attending the MDT were not the least qualified professionals but they were certainly the group who had the most day-to-day contact with the service user.

In line with the results from Cameron and Lart (2003)’s and Nic a Bháird *et al.* (2016) systematic reviews, this study suggests that clear aims and objectives are a pre-requisite to team working but, in addition, there needs to be an understanding of how those aims and objectives can be implemented. While there was agreement that each team member had the responsibility for decision making and that everyone had a professional obligation to speak out during meetings, the observations revealed a number of barriers to this taking place in practice.

Perceptions about sharing decision making with the service users in the interviews with staff did not match the reality of the actual meetings. While the MDT meetings were viewed as a forum for discussion with the service users, these were only invited to the meeting after all of the staff had presented their reports. This is not surprising, given the longstanding tradition of psychiatric paternalism in mental health care, and health workers potentially being unwilling to trust and respect the service user view (Hansen *et al.*, 2004). Although in recent years there has been a clear move within mental health services to promote service user choice and self-determination, this is sometimes at odds with forensic services’ need to manage risk to others (Pouncey and Lukens, 2010), reaffirming the ongoing tension between risk/security and care/therapeutic activities within secure services (Davies, 2004). To resolve this conflict, services try to involve the service user in their care plan in partnership with the MDT (Mann *et al.*, 2014). Results from the observations in this study indicate that, although service users get involved in their care plan, there is little continued engagement or understanding of how to achieve that plan. Service users need to be encouraged to have a more vested interest in their recovery throughout the duration of their stay, and understand every discipline’s role in their recovery.

True multidisciplinary collaboration involves people working together towards the same goal and a shared understanding of an issue. However, as argued in this paper, the decisions are unequally shaped by the professional and personal values and assumptions of those involved, as well as by the power dynamics linked to the knowledge and (legal) responsibility of each member of the team. Coles (2012) argues that “professionals and service users can have assumptions, values and goals that are in conflict with each other”. Research should be conducted to further understand the way in which these differences can be reconciled; and the extent to which professionals understand the service user’s perspective. Further ethnographic research should explore the nuances of interactions between MDT professionals and service users during the meetings, the language used and the approach taken by professionals to enable/empower service user to participate in the decision-making process.

### Limitations

The following limitations should be considered, when interpreting the results presented here. Only a small proportion of MDT meetings were observed over a relatively short period of time. Although some of these meetings were consecutive and documents were additionally analysed to account for any gaps between meetings, discussions and decisions team members made outside of the meetings could not be accounted for and these may have explained some of the inconsistencies observed.

The quality of the videos was directly linked to the quality of the MDT meeting rooms. Often there was little space to work with the equipment and small rooms meant that some team members’ body language and presentation during the meeting were missed. The presence of the video equipment and the researchers could have led to team members and service users acting less naturally. This could have affected when and how people chose to contribute to discussion, perhaps by monitoring language or not speaking up. However, direct observations enabled unmediated access to how people conducted themselves in meetings rather than having to rely only on people’s subjective accounts (from the interviews).

## Concluding comments

This study is unique in using direct (and video recorded) observations in secure mental health settings to explore the challenges of decision making in a multidisciplinary format. The study contributes to the literature on multidisciplinary working in several ways. Evidence on how MDT meetings in forensic settings function in practice is limited. Comparing private and public accounts of the MDT meetings, this study has attempted to understand some of the inter- and intra-professional cultural or power issues in these highly complex settings. Although limited, the emerging evidence does point to the challenges of achieving an equally balanced/democratic decision-making process, especially with regards to shared decision making, where service users’ values and input should also be considered. One could argue that, given the way mental health services are structured and governed (under the Mental Health Act 2007), full collaborative decision making is more illusory or even tokenistic than actual. Regardless, giving service users greater responsibility and power in the process should continue to be an aim.

## Figures and Tables

**Table I:** “Old” MDT meetings vs “New” MDT meetings

Decision-making forum	Decision-making forum
No official chair; RC’s responsibility to manage the meeting and discussion	Independent chair (not the RC, not part of the patient’s current care team, nursing/clinical background); 2 chairs for the 4 teams
All service users discussed weekly (up to 18 service users per RC)	Each service user discussed fortnightly (up to 9 service users per RC)
Lengthy meetings; attendance fluctuated between and in each meeting; little time for SUs discussed towards the end Unpredictable meeting times which took place on ward; “ward on hold” (waiting for the team to arrive)	Shorter meetings; consistent attendance; more set time for each SU (approx. 30 min) Fixed meeting times in a set room on the ward or a suitable office meeting room within the clinic (outside the ward)
Not all disciplines involved/contributing Minutes less structured	All disciplines must participate by producing a summary prior to the meeting (Care Review Form) More accountability (transparency?) – minutes recorded for each meeting (including an “action plan” box and care review summary for each service user); attendance and summaries audited
Informal, no predetermined structure, no action log	Formal, structured, minutes and action log, better time management; summary form
Less opportunity for SUs to attend; not invited to attend; SU rarely attended	All SUs asked to attend; SU more likely to attend now
No systematic risk formulation Decisions based on unsystematic assessment of current presentation (stable)	Active risk formulation; more drive on index offence and psychology work Evidence based – considering risk, previous risk, current presentation
RC on leave – no meeting	Meeting takes place regardless (although no “legal” or key decisions made if RC not present)
